# Supplementary material for: The zinc-finger transcription factor Sfp1 imprints specific classes of mRNAs and links their synthesis to cytoplasmic decay
Source: eLife. 2024 Oct 2;12:RP90766. doi: 10.7554/eLife.90766 (PMC11446548; doi:10.7554/eLife.90766)
Supplement: Supplementary file 1. [file elife-90766-supp1.pdf]

Table S1

Yeast strains

BY4741	*MAT a his3Δ1 leu2Δ0 met15 Δ0 ura3 Δ0*	EUROSCARF
Y05312	BY4741 *ylr403w (sfp1)::*kanMX4	EUROSCARF
LMY3.1	BY4741 *YJL140w (RPB4)::*MYC18-TRP1	This work
LMY7.1	BY4741 YJL140w (RPB4)::Myc18-TRP1 ylr403w(sfp1)::kanMX4	This work
yMC1019	BY4741, HTP-*SFP1*	This work
yMC1020	BY4741, HTP-*SFP1*	This work
yMC1021	BY4741, HTP-*SFP1 rpb4Δ::*CloNAT	This work
yMC1022	BY4741, HTP-*SFP1 rpb4Δ::*CloNAT	This work
yMC189	MATalfa, *his3 leu2 ura3 ade2 ade3 nup49::TRP1* pUN100 (CEN/*LEU2/nup49-313)*	P. Silver (PSy413)
yMC190	MATa *rpb1-1 his3 leu2 ura3 ade3 nup49::TRP1* pUN100 (CEN/*LEU2/nup49-313*)	P. Silver (PSy844)
yMS1	yMC189 + pMC295 (pRS316::GFP-*SFP1*)	This work
yMS2	yMC190 + pMC295 (pRS316::GFP-*SFP1*)	This work
yMS119	*leu2 ura3 rpb4*Δ::KAN, *sfp1Δ*::*ura3* + pMC305 (pRS316::*RPB4*-RFP)+ pMC295 (pRS315::GFP-*SFP1*)	This work
yMC805	W303 background; *leu2-3,112 HIS3,15 ura3-1*::p*ADH1*-OsTIR1-*URA3 ADE2 trp1-1* can1-100 MATα	David Shore
yMC806	W303 background; *SFP1*-AID::hph, leu2-3,112 HIS3,15 ura3-1::pADH1-OsTIR1-URA3 ADE2 trp1-1 can1-100 MATα	David Shore
